# Implementing open science and reproducible research practices in mental health research through registered reports

**DOI:** 10.1002/jcv2.12275

**Published:** 2024-09-02

**Authors:** Jessie R. Baldwin, Henrik Larsson

**Affiliations:** ^1^ Department of Clinical Educational and Health Psychology University College London London UK; ^2^ School of Medical Sciences Örebro University Örebro Sweden

**Keywords:** mental health research, open science, registered reports, reproducible research

## Abstract

To increase the number of pre‐registered observational studies, *Journal of Child Psychology and Psychiatry (JCPP) Advances* is delighted to now invite Registered Reports. Registered Reports are a format of article in which the study protocol is pre‐registered and peer‐reviewed before the research is conducted. If the protocol is of high quality and the proposed research topic is important, *JCPP Advances* will commit to publishing the study regardless of the results. This article format crucially addresses publication bias, as decisions on publication are entirely independent of the results.

## EDITORIAL

The value of open science and reproducible research has been increasingly recognized in child and adolescent mental health research, where transparency and reproducibility are crucial for building a reliable evidence base. Open science principles focus on the unrestricted sharing of data, code, materials and publications, enabling others to freely access and build upon existing research. Reproducible research complements these principles by ensuring that research findings can be independently verified and validated. Key aspects of reproducible research include pre‐registration of study protocols to prevent selective reporting, comprehensive reporting of methods and results to ensure clarity and transparency, the use of large sample sizes to enhance the reliability of findings, and the promotion of replication studies to confirm the robustness of results.

The Journal of Child Psychology and Psychiatry (JCPP) Advances have already implemented several open science and reproducible research initiatives, such as requiring data availability statements, offering open science badges and organizing special issues with a focus on ‘open science and reproducible research’ relevant topics. For example, a recent special issue in JCPP Advances, published in December 2023, focussed on high‐quality evidence‐synthesis studies (Bellato et al., 2023). Such studies contribute to reproducible research by providing a transparent, systematic approach to reviewing and combining evidence, identifying biases, and offering insights that help ensure future research is more robust and reliable. The next special issue of JCPP *Advances* is scheduled for late 2024 and will focus on participatory research, which is an important component of open science as it involves collaboration with stakeholders such as patients, families, and communities throughout the research process, ensuring that studies are relevant, ethical, and impactful.

Recent editorials and editorial perspectives published in JCPP Advances have contributed to an increased understanding of open science and reproducible research practices by highlighting the value of using large samples sizes and transparent reporting (Larsson, 2022), as well as by clarifying how an increased meta‐research literacy may ensure high quality, transparent and reproducible primary data or meta‐research products (Fabiano et al., 2024).

Several papers in the September issue of JCPP Advances provide useful examples of good open science and reproducible research practices. Six papers in the current issue used very large sample sizes based on data from the Swedish twin registry (Garcia‐Argibay et al., 2024; Keijser et al., 2024), the Prediction and Prevention of Preeclampsia and Intrauterine Growth Restriction (PREDO) study (Lahti‐Pulkkinen et al., 2024), the UK's Millennium Cohort Study (Tsomokos et al., 2024), and large‐scale national registers or health care databases (Coughlan et al., 2024; Nordmo et al., 2024). Two papers (Donaldson, Hawkins et al., 2024; Tsomokos et al., 2024) in the September issue of JCPP Advances made the data openly available (in both studies via the UK Data Service) and one paper made the code that was used to generate correlations, models and figures publicly available on their GitHub page (Keijser et al., 2024).

Today, most publications that report findings from clinical trials or systematic reviews are based on pre‐registration of study protocols (indeed, this is mandatory for clinical trials). In contrast, pre‐registration is much rarer for studies using observational data. For example, none of the publications in the September issue of JCPP Advances were pre‐registered. To increase the number of pre‐registered observational studies, *JCPP Advances* is delighted to now invite Registered Reports. Registered Reports are a format of article in which the study protocol is pre‐registered and peer‐reviewed before the research is conducted. If the protocol is of high quality and the proposed research topic is important, *JCPP Advances* will commit to publishing the study regardless of the results. This article format crucially addresses publication bias, as decisions on publication are entirely independent of the results.

## HOW DO REGISTERED REPORTS WORK?

The process of conducting a Registered Report occurs in two stages. In the first stage, authors submit their study protocol outlining the background literature, research question(s), hypotheses, and proposed methods. This will be assessed by peer reviewers, who will evaluate the importance of the research question and quality and robustness of the methods. The article will then either be accepted in principle for publication, revised, or rejected. Following acceptance in principle, authors will proceed to the second stage, in which they conduct the study adhering to their peer‐reviewed protocol. They will write up their manuscript including the results and discussion, and submit this manuscript for Stage 2 peer‐review. If the authors have followed their protocol, labelled any extra analyses as post‐hoc, and interpreted their findings sensibly, the manuscript will be published. In addition to preventing publication bias, Registered Reports thus help to address other questionable research practices (e.g., p‐hacking, selective reporting, and hypothesising after the results are known), by ensuring that authors adhere to their pre‐registered protocol.

## THE VALUE OF REGISTERED REPORTS IN MENTAL HEALTH RESEARCH

Registered Reports are particularly valuable in mental health research, given widespread evidence of publication bias and questionable research practices, which can distort the evidence base (Baldwin, 2024). For example, studies have found that almost all (98%) positive trials for antidepressants get published, relative to only 48% of negative trials (De Vries et al., 2018). This is also the case for psychotherapy trials, where published papers show an excess of statistically significant findings (Flint et al., 2015). Furthermore, questionable research practices (e.g., p‐hacking, selective reporting, and hypothesising after the results are known) have been found to be particularly common in psychology and psychiatry relative to other fields (Head et al., 2015; Rubin, 2017).

Meta‐scientific evidence suggests that Registered Reports are effective in reducing publication bias and such questionable research practices. For example, 60% of Registered Reports reported null results, which is five times greater than the rate in regular articles (Allen & Mehler, 2019). Another study found that over half (56%) of Registered Reports reported findings that did not support the primary hypothesis, compared to just 4% of regular studies (Scheel et al., 2021).

Interestingly, evidence also suggests that Registered Reports are rated as higher quality than regular articles. For example, a sample of Registered Reports from psychology and neuroscience were rated as higher in methodological rigour, analytical rigour, and overall quality than regular articles matched for topic, author and journal (Soderberg et al., 2021). Greater rigour and study quality in Registered Reports may be due to study acceptance being driven by high quality study design, rather than positive study results, as in typical articles.

## CONCLUSIONS AND FUTURE DIRECTIONS

Considering the interest of *JCPP Advances* in open science and reproducible research in the field of child and adolescent mental health, we would like to suggest the following good practices that we encourage research teams to follow, when preparing studies based observational data (e.g., prospective cohort studies, register studies) for submission to this journal.

- Pre‐register study protocols and/or conduct research as a Registered Report, to help protect against questionable research practices and publication bias
- When preparing a Registered Report, ensure that your study addresses an important research question, using high quality, robust methods. Describe the methods in sufficient detail to allow replication and prevent undisclosed flexibility in the analysis.
- Make analytic code openly available on GitHub or the Open Science Framework
- When possible, make data and materials openly available.
- Consider collaboration with relevant stakeholders such as patients, families, and communities with lived experience throughout the research process to ensure that studies are relevant, ethical, and impactful.
- Consider using relevant reporting guidelines such as Strengthening the Reporting of Observational Studies in Epidemiology (STROBE) ‐ developed by the EQUATOR Network for the reporting of observational research.

## AUTHOR CONTRIBUTIONS

**Jessie R. Baldwin**: Writing – original draft; writing – review & editing. **Henrik Larsson**: Writing – original draft; writing – review & editing.

## CONFLICT OF INTEREST STATEMENT

Henrik Larsson reports receiving grants from Shire Pharmaceuticals; personal fees from and serving as a speaker for Shire Pharmaceuticals and Evolan Pharma AB outside the submitted work; and sponsorship for a conference on attention‐deficit/hyperactivity disorder from Shire Pharmaceuticals outside the submitted work. Henrik Larsson is Editor in Chief of JCPP Advances. Jessie Baldwin is Co‐Editor for Registered Reports at JCPP Advances.

## ETHICAL CONSIDERATIONS

None.
